# An analysis of the key drivers of the Japanese digital therapeutics patents: A cross‐sectional study

**DOI:** 10.1002/hsr2.1268

**Published:** 2023-06-01

**Authors:** Tetsuaki Oda, Chikako Oda

**Affiliations:** ^1^ Department of Management of Technology Ritsumeikan University Osaka Japan; ^2^ Department of Medical Information and Life Sciences, Graduate School of New Areas The University of Tokyo Tokyo Japan

**Keywords:** digital therapeutics, DTx, key driver, patent analysis, patent citations

## Abstract

**Background:**

Digital therapeutics (DTx) are software or other tools that support or implement medical practices such as disease prevention, diagnosis, and treatment using digital technology. DTx has been approved in Japan, and it is anticipated that the number of approvals will increase in the future. DTx differs from conventional medical devices in that its primary purpose is treatment.

**Aim:**

This study aims to identify the key drivers of DTx in Japan by analyzing patents in the field of medical information, including DTx.

**Methodology:**

This study visualizes the results of patent analyses for DTx and examines patent applications that feature applied technology and indications in the medical information field as the key drivers. The study will also employ patent citation analysis. It can be argued that the more citations a patent receives, the more similar research and development activities are being conducted, and the greater the competition. The number of citations per patent application will also be calculated to help identify areas where the value per patent application is high and competition intensifies. A patent citation matrix analysis will be conducted for notable Japanese companies in the DTx field. The citation matrix analysis consists of the number of citations and the company's selfcitation ratio to visualize the patent value. This study investigates the key drivers of DTx by analyzing patent technologies, focusing on patent applications with a high number of citations or a high selfcitation ratio.

**Results:**

Key drivers of digital therapeutics were examined by analyzing patents in the fields of healthcare informatics and diagnostics. In terms of the number of patent applications and citations in Japan, numerous patents were related to “applications,” “sensors,” “medical imaging,” “central nervous system/psychiatry,” and “heart.” As a result, Japanese companies are expected to conduct R&D with an eye toward overseas expansion.

## 1 INTRODUCTION

Digital Therapeutics, also known as DTx, are software or other tools that support or implement medical practices such as disease prevention, diagnosis, and treatment using digital technology.

On June 19, 2020, Cure‐Up and Keio University School of Medicine developed an antismoking system that includes an analyzer to measure carbon monoxide concentration in exhaled breath and a medical device program that assists smoking cessation treatment by encouraging behavioral changes. The company was approved to manufacture and sell the system as a medical device. Other applications include a platform to improve the lifestyle of diabetes patients (Mitsubishi Tanabe Pharma Corporation), an application to treat major depressive disorder (MDD) (Otsuka Pharmaceutical/Click Therapeutics), an application to treat attention deficit hyperactivity disorder (ADHD) (Shionogi Pharmaceutical Co./Akili Interactive Inc.), and others in development.

In addition, related patents have been filed globally to protect the intellectual property resulting from the development of these digital therapies.

The purpose of this study is to examine the key drivers of digital therapy by analyzing patents in the fields of healthcare informatics and diagnostics, including digital therapy (medical information field). The key drivers are defined as keywords promoting the development of DTx. The key drivers of digital therapy will be extracted in terms of applicable technologies and indications in the medical information field.

## 2 METHOD

The key drivers are defined as keywords promoting the development of DTx. The key drivers of digital therapy will be extracted in terms of applicable technologies and indications in the medical information field using the International Patent Classification (IPC) of 2021, which creates a hierarchical system of language‐independent symbols for the classification of patents and utility models according to different technical fields. We collected patent data using the patent database provided by Panasonic and identified existing DTx technologies and candidate technologies for DTx. Even if patents have common IPC codes, they can be classified into similar technologies. Based on text information, we extracted the keywords of DTx technologies and candidate technologies associated with each keyword.

**Figure 1:**
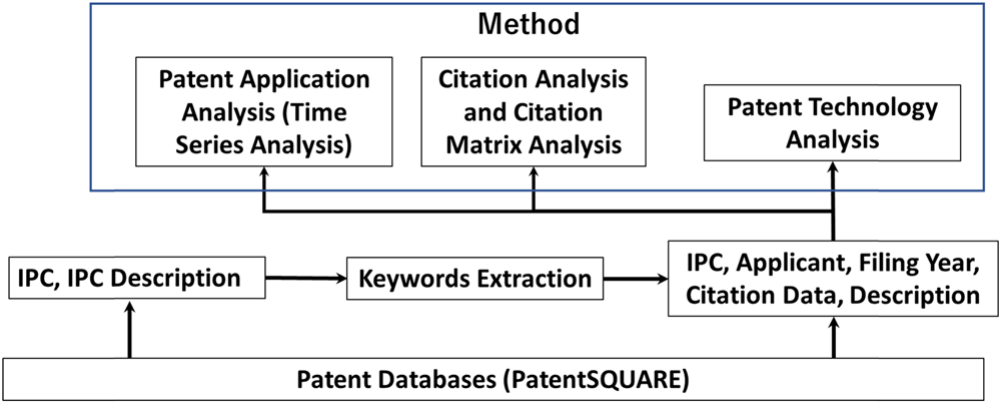
Methods and process of the analysis.

As shown in Figure 1, the methods of Patent Application Analysis (Time Series Analysis), Citation Analysis, and Patent Technology Analysis are employed in this study through keyword extraction based on data from IPC, IPC description, applicant, filing date, citation, and patent description of patent databases.

### 2.1 Patent application analysis (time series analysis)

This study will visualize the results of the patent analysis of digital therapy and analyze patent applications in the field of medical information, using applied technology and indications as key drivers to examine the key drivers of digital therapy. The visualization of the patent analysis will be conducted through quantitative analysis based on the patent information collected from the patent database. The patent database, provided by Panasonic, contains patent information published since 1971.^1^ Furthermore, by analyzing information on patent applicants, it is possible to organize the rights relationship in joint research and development between medical institutions and companies.

### 2.2 Citation analysis and citation matrix analysis

In this study, Patent Citation Analysis is also used. Patent citation analysis is a method that assumes that the higher the number of citations, the higher the value of the patent; however, it can also be said that the higher the number of citations, the greater the number of similar research and development projects, and the more intense the competition. The number of citations per patent application is also calculated. This will identify areas where the value per patent application is high and competition is intense.^2^

In addition, a patent citation matrix analysis will be conducted on Japanese companies of interest in digital therapy. This is an application of the Boston Consulting Group's Product Portfolio Matrix (market growth and market share matrix) to the evaluation of patent value, and is composed of the number of citations (growth potential of patent value) and the company's citation ratio (technology share by the company), and can visualize patent value.^1^

In this study, we extracted patent applications for digital therapy in Japan using the patent database and show trends in patent technology through citation and citation analysis.

### 2.3 Patent technology analysis

This study will examine the key drivers of digital therapy by extracting characteristic patent applications through time series analysis, citation analysis, and citation matrix analysis, and analyzing patent technology by focusing on patent applications with a high number of citations or in‐house citation ratio. To conduct a patent technology analysis, the applicant, title of invention, and abstract of the patent application will be presented.

## 3 RESULT OF THE ANALYSIS

### 3.1 Patent application analysis (time series analysis)

**Figure 2:**
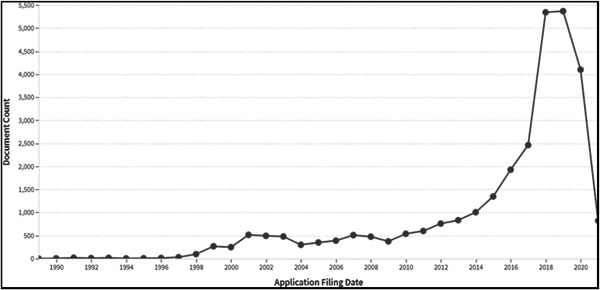
The number of patent applications indicating IPC “G16H” and “A61B.”

To facilitate international use, patent documents are classified according to the IPC. The IPC analysis is conducted by extracting patents from databases and analyzing them statistically. The IPCs for technical fields that include digital therapy are “G16H” and “A61B.” Here, IPC “G16H” is assigned to “Healthcare Informatics, that is, medical or health care information and communication technology particularly adapted to the handling or processing of data.” IPC “A61B” includes “diagnosis, surgery, personal identification.” A patent analysis website was used for the survey. The number of patent applications in the technical field (medical information field) corresponding to IPC “G16H” and “A61B” totaled 30,933. Figure 2 shows the number of patent applications in the medical information field as of September 10, 2021. The horizontal axis represents the priority date (or the original filing date if priority was claimed), and the vertical axis represents the number of patent applications worldwide. Figure 2 shows that the number of patent applications has been increasing since 2015, reaching 5368 in 2019. The number of patent applications in 2020 and 2021 decreased due to the time lag until the publication of patent applications.

**Figure 3:**
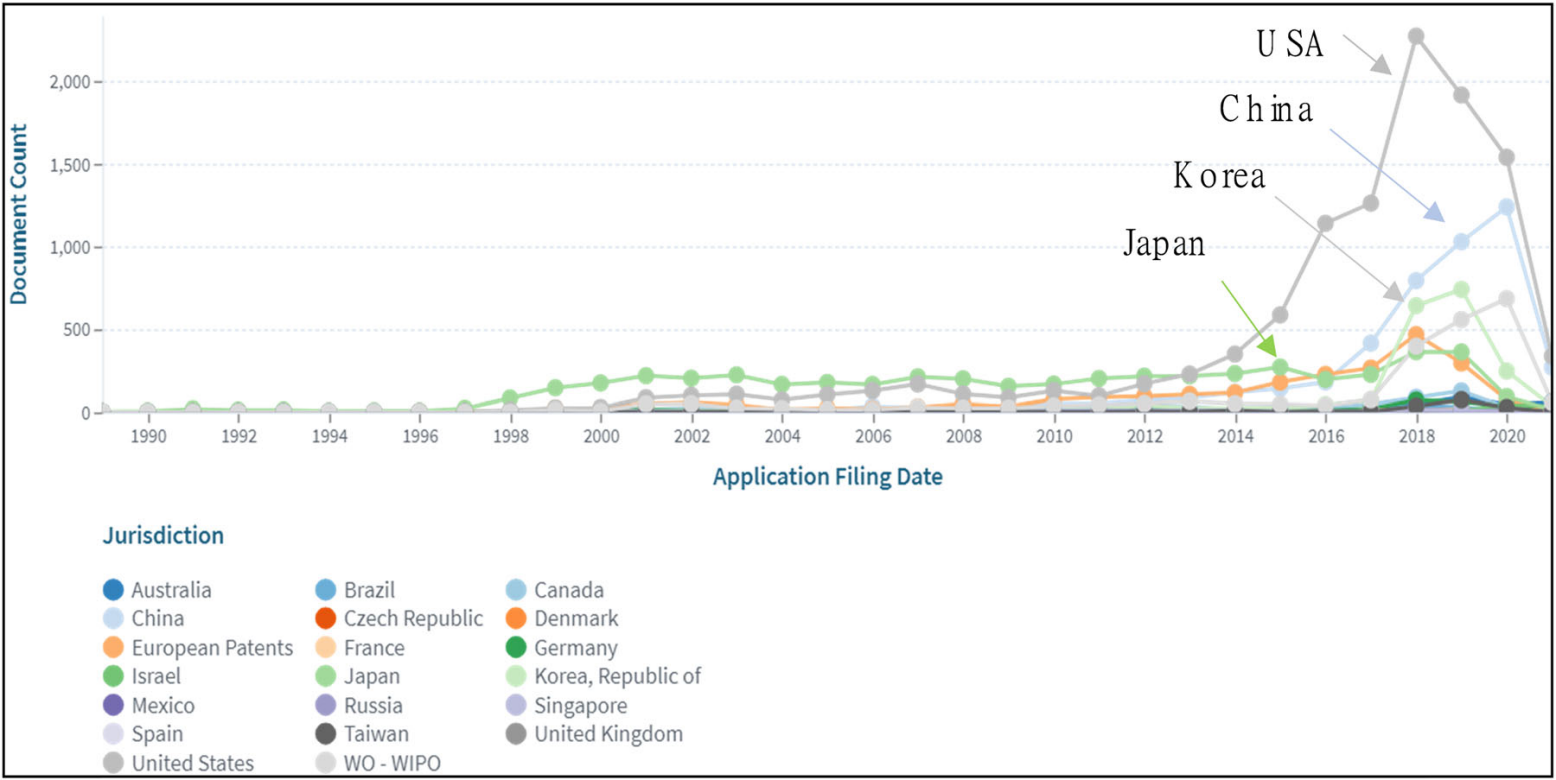
Number of patent applications indicating IPC “G16H” and “A61B” by countries.

Figure 3 shows the number of companies classified by country. By country, Japan ranked fourth, following the United States, China, and South Korea, excluding WO by Patent Cooperation Treaty and European Patent.

**Figure 4:**
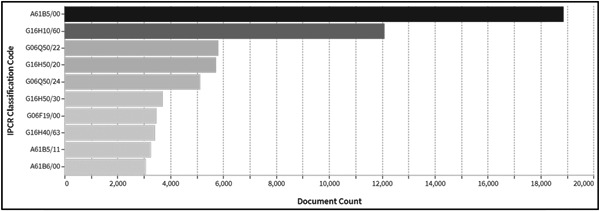
Number of patent applications per International Patent Classification (IPC).

Figure 4 shows the top 10 assigned IPCs. The horizontal axis represents the number of patent applications. Table 1 is a table showing the represented IPCs “G16H,” “A61B,” and others. If the technology field of the invention covers a wide range, more than one IPC may be assigned. IPCs other than “G16H” and “A61B” are also extracted in Figure 4. The IPCs are subdivided into more precise sections (Subclass). “A61B 5/00” indicates “Detection, measurement, recording, and identification for diagnosis.” “G16H 10/60” indicates “Patient‐specific data.” “G06Q 50/22” indicates “Social welfare services.” “G16H 50/20” indicates “Computer‐aided diagnosis.” “G06Q 50/24” indicates “Educational management or guidance.” “G16H 50/30” indicates “Calculating health indicators, Individual health risk assessment.” “G06F 19/00” indicates “Digital computing, Data processing apparatus or methods, Special applications.” “G16H 40/63” indicates “Local operation.” “A61B 5/11” indicates “Measuring movement of the whole body or parts thereof,” and “A61B 6/00” indicates “Equipment for radiological diagnosis.”

**Table 1:** Description of International Patent Classification (IPC).

IPC (Subclass)	Description
A61B 5/00	Detection, measurement, recording, and identification for diagnosis
G16H 10/60	Patient‐specific data
G06Q 50/22	Social welfare service
G16H 50/20	Computer‐aided diagnosis
G06Q 50/24	Educational management or guidance
G16H 50/30	Calculating health indicators, individual health risk assessment
G06F 19/00	Digital computing, data processing equipment or methods, special applications
G16H 40/63	Local operation
A61B 5/11	Measuring movement of the whole body or parts thereof
A61B 6/00	Equipment for radiological diagnosis

Figure 4 is a graph showing the number of patent applicants. The vertical axis indicates the number of patent applications. The top patent filers include “Philips,” “Toshiba,” “Fujifilm,” “Siemens,” “Canon,” “Samsung,” and others. Note that “Toshiba” and “Toshiba Medical Systems” are counted as different applicants.

### 3.2 Time series analysis and citation analysis of patent applications

#### 3.2.1 Keyword extraction

In addition to the above IPCs, keywords are extracted to analyze patents containing specific keywords. The extraction of keywords utilizes the F‐term, a technical classification unique to the JPO that focuses on the technical feature points of literature and is developed with computer searches in mind. First, major keywords were selected from the technical aspect of digital therapy and the indication or treatment site aspect. Then, explanations of F‐terms containing the major keywords were searched, and minor keywords (subterms) were selected from the retrieved explanations. Table 2 shows the major and minor keywords that were extracted.

**Table 2:** Extracted keywords.

Major term of keywords	Subterm of keywords (Search fomula)
Artificial intelligence	Microcomputer, artificial intelligence, machine learning, deep learning, neural network, expert system, knowledge base, artificial brain
Application	Blog, SNS, membership, participatory+social, review, communication tool, application
Biodata	Biometric data, safety confirm, whereabout, behavior, life response, biological reaction, vital
Prediction	Prediction, predicted estimated, planned disease
Database	Big data, medical records, diagnostic data
Remote	Remote
Robot	Robot, android, robotics, humanoid
Sensor	Sensor
Medical image	CT, imaging, magnetic resonance, nuclear medicine, endoscopy, MRI, medical imaging
Image processing	3D imaging, image intensifier, image processing, image processing
Diabetes	Diabetes, ketone, metabolism, gout, glymidinesodium, glypizaid, fenforminhydrochloride, buformin hydrochloride+metformin+aprinol+antihabit, insulin, carbohydrate, blood sugar, ketosis+ketoacidosis
Obesity	Obesity+diet, calori, aspartame, interleukin, P‐cell stimulating factor, PCSF+multi colony stimulating factor, B cell stimulating factor, B cell growth factor, BCGF+appetite, weight loss agent
Heart	Heart, ECG, heart beat, cardio pulmonary, blood test, needle electrode, insertion electrode, pacemaker, circulator, atrial, ventricle, mitral, tricuspidvalve, inotropic+heart failure, heart muscle, hematopoietic, electrical potential map, electrode catheter, diaphragm, arrhythmia, balloon pump, IABP + UCG
Blood vessel	Vascular, vein, arteries, pulse wave, pulse, blood pressure, cardiac pressure, blood flow, heart beat, pulsation+korotkoff sound, k sound, hemostasis+manchette, cuff, compression band, blood clot, hemostasis, catheter, blood collection, glycolysis+bleeding, cholesterol, coronary dilatation, antihypertensive agent, suture, bandage, plaster cast, pseudo‐endometrium, heparin, chondroitin, blood circulation, serum, plasma, neurotensin, fibroblast, blood+circulatory system, hemophilia, blood stasis, platelet, basophil, eosinophil, macrophage, thomascell, lymphocyte, angiography, flow imaging, forcep, bypass surgery, guide wire, stent, vascular, anti‐PAF
Central nervous system and psychiatry	Mental, nerves, emotion, cognition, stimulatinghormones, perception, dopamine, neurons, neuropathy, cerebral, cerebellum, medullaoblongata, anti‐rigidity, psychotropics, anesthesia, sleep, sedation, convulsions, analgesic, antifebrile, central, anxiety, insomnia, anguish, pain, hyperreflexia, excitement, synapse, disorientation
Digestive organs	Centralnervous system, digestivesystem, stomach, ulcer, intestine, pancreas, liver, digestion, esophagus, abdomen, anus, buttocks, buttocks, hemorrhoids, largestool
Respiratory organs	Respiration, lung, residualairvolume, cough, nasalsound, respiratoryresistance, respiratoryimpedance, nasalcavity, airway, thorax, thoraciccage, nasalpassage, bronchi, asthma, phlegm, tuberculosis, emphysema, atomizer
Ophthalmology	Ophthalmology, ocular, fundus, eyeexamination, modeleye, eyewidth, iris, iris, cornea, eye, intraocularpressure, cataract, eyedrop, eyeointment, gaze, frenzel, glasses, glasses, eyetracking
Cancer/tumors	Cancer, tumor, anticancer, anticancer, TNF, CEA, src, myc, mutagens, carcinogenesis, carcinogenesis, tumornecrosis, hematoporphyrin, HPD, immunogenicity, cytokine, immunity, monokines, lymphokine, thymosin, thymosin, interleukin, interleukin, LAF, interleukin, muramyldipeptide, interferon, IFN, INF, necrosis, normalcells, abnormalcells, bleomycin, decalcification, de‐cancer, fibroblast, oncovirus, ascites, malignant, leukemia, radiationtherapy, hyperthermia, phosphotoxin, photoimmunity, photosensitivity
Nose and ears	Ear, ear, nose, throat, sensoryorgans, hearingloss, rhinitis, otitis, parotidgland
Oral cavity	Oral cavity, gargle, pharynx, lips, inthemouth, badbreath, throat, sublingual, swallowing
Dentistry	Dental, dentistry, dentaladhesive, handpiece, rinsing, toothbrush, toothbrush, cleaningequipment, cutting, drilling, straightening, jaw, denture, dentalmirror, artificialteeth, implant, occlusion, alveolar, ceramic, prosthetic, dentalcrown, crowns, mouthwash, mouthwash, mouthwash, badbreath, dentalplaque, toothroot, periodontal, toothdecay, enamel, toothpaste, toothpaste, anaerobicadhesives, resin, amalgam
Radiation therapy	Radiation therapy, X‐ray therapy, Nuclear medicine
Electrical stimulation	Radiation therapy, electrical stimulation, electric pulse, action potential, low frequency therapy, prestimograph, flicker, psychological response, electromyography
Addictive behavior	Addictiveintoxication, opium, narcotics, alcohol, smoking cessation, no smoking, smoking, abstinence from alcohol, no drinking, drugs
Pain	Pain, pack pain, neuralgia, blood circulation enhancement, acupuncture points, soothing, sedation
Allergy	Allergy, auto immunedisease, steroids, anaphylaxis, PAF, platelet‐activating factor, skin reaction test, patch test, cellular immunity, histamine, asthma, rhinitis
Infectious disease	Infectious disease, bacteria, viruses, viruses, reverse transcriptase inhibition, antiphage, antireplicase, herpes, antibacterial, antimicrobial, parasite, fungus
Taking medicine	Medication, guidancenote

Frequently occurring words in patent applications assigned IPCs “G16H” and “A61B” are shown in Table 3. These frequently occurring words were also used as references for the extraction of subterm keywords.

**Table 3:** Frequently used words.

Frequent ranking	Frequent word	Frequent ranking	Frequent word	Frequent ranking	Frequent word
**1**	STEP	**34**	MEDICAL INSTITUTION	**67**	MEDICAL IMAGING
**2**	MEDICAL IMAGE	**35**	IMAGE PROCESSING DEVICE	**68**	MEDICAL DATA
**3**	BIO INFORMATION	**36**	DEVICE	**69**	RELATED INFORMATION
**4**	INFORMATION TECHNOLOGY DEVICE	**37**	DISPLAY CONTROL UNIT	**70**	ALERT
**5**	REPORT	**38**	EVENTS	**71**	COMPUTING DEVICES
**6**	TERMINAL EQUIPMENT	**39**	STRESS	**72**	LEARNING MODEL
**7**	ASSISTIVE DEVICE	**40**	MEDICAL IMAGES	**73**	IMAGE PICK‐UP DEVICE
**8**	PATIENT INFORMATION	**41**	DIAGNOSTIC DEVICE	**74**	ALARM
**9**	USER TERMINAL	**42**	CLIENT	**75**	CATEGORY
**10**	PROCESSING CIRCUIT	**43**	PATIENT DATA	**76**	TRAINING
**11**	MANAGEMENT UNIT	**44**	MEASURING DEVICE	**77**	PREDICTION MODEL
**12**	DATABASE	**45**	ICON	**78**	HEALTH INFORMATION
**13**	HEALTH STATE	**46**	SENSOR DATA	**79**	MEDICAL EQUIPMENT
**14**	DATA SET	**47**	USER INTERFACE	**80**	SURGICAL INSTRUMENTS
**15**	ELECTRONIC MEDICA RECORD	**48**	USER ID	**81**	NORMAL RANGE
**16**	INFORMATION ACQUISITION	**49**	REPORTING	**82**	PICTURE DISPLAY DEVICE
**17**	MANAGEMENT UNIT SERVER	**50**	MEDICAL INFORMATION	**83**	NEURAL NETWORK
**18**	PROCESSOR	**51**	STORAGE CIRCUIT	**84**	FIELD
**19**	MEDICAL IMAGE DATA	**52**	INSPECTION IMAGE	**85**	MODULE
**20**	MODALITY	**53**	ENGINE	**86**	INPUT SCREEN
**21**	MACHINE LEARNING	**54**	DATA ACQUISITION	**87**	INFORMATION STORAGE
**22**	SERVER	**55**	REGION OF INTEREST	**88**	TEACHER DATA
**23**	INSPECTION INFORMATION	**56**	SYSTEM	**89**	INSPECTION RESULTS
**24**	IMAGE DATA	**57**	MEDICAL INFORMATION PROCESSING	**90**	DIAGNOSTIC RESULT
**25**	CLOUD	**58**	MEASUREMENT DATA	**91**	GAS CONCENTRATION
**26**	INFORMATION PROCESSING SYSTEM	**59**	ENVIRONMENTAL INFORMATION	**92**	COMPOSER
**27**	BIODATA	**60**	VITAL SIGNS	**93**	MONITORING
**28**	PORTABLE TERMINAL	**61**	INFORMATION TERMINAL	**94**	ENDOSCOPY
**29**	SUPPORT SYSTEM	**62**	CORTISOL	**95**	PHOTOGRAPHIC EQUIPMENT
**30**	DIAGNOSTIC AID	**63**	SERIES	**96**	INSPECTION DEVICE
**31**	WEARABLE	**64**	RECORD	**97**	INSULIN
**32**	CLIENT TERMINAL	**65**	IDENTIFICATION INFORMATION	**98**	MODULE TYPE
**33**	USERS	**66**	DATA ANALYSIS	**99**	USER INTERFACE
				**100**	HEALTH CARE

IPC “G16H” and “A61B” as well as the above‐mentioned subterm keywords in the abstracts were searched, and the results are presented separately for the time series analysis of patent applications and the analysis of citations. In the time series analysis, the number of patent applications for each major keyword is displayed and analyzed by year (year of filing). In the citation analysis, the number of citations for each major keyword is displayed and analyzed by year. The search was conducted from September 1st to 10th, 2021.

#### 3.2.2 Time series analysis of Japanese patent applications

Tables 4, 5, 6, 7 show the number of patent applications in a time series. The largest number of patent applications was for medical imaging (295 cases), followed by prediction (183 cases), sensors (164 cases), central nervous system/psychiatry (161 cases), bio‐data (151 cases), sleep (98 cases), image processing (76 cases), vascular (66 cases), artificial intelligence (64 cases), cardiac (44 cases), remote (35 cases), diabetes (32 cases), apps (29 cases), respiratory (28 cases), ophthalmology (28 cases), genitourinary (23 cases), big data (20 cases), addiction (15 cases), dental (11 cases), VR/AR (11 cases), cancer/oncology (10 cases), breast (7 cases), oral (7 cases), medication (6 cases), robotics (6 cases), gaming (5 cases), genetic (5 cases), gastrointestinal (4 cases), obesity (4 cases), diet (3 cases), allergy (3 cases), infection (3 cases), and pain (1 case). The number of other patent applications was zero. The number of patent applications has been increasing rapidly since around 2012, especially since 2015.

**Table 4:** Time series analysis (medical image to image processing).

Filing year	Medical image	Prediction	Sensor	Central nervous system and psychiatry	Biodata	Sleep	Image processing
2006	1	0	0	0	0	0	0
2007	0	0	1	0	0	0	0
2009	1	0	0	0	0	0	0
2011	1	1	0	0	0	0	0
2012	1	0	1	1	0	1	1
2013	10	1	2	0	3	0	2
2014	21	3	10	6	1	4	6
2015	33	11	3	7	15	4	4
2016	33	6	7	8	12	3	9
2017	29	24	29	15	20	7	10
2018	60	37	54	45	35	29	15
2019	74	64	40	51	40	30	20
2020	27	35	15	28	23	20	7
2021	4	1	2	0	2	0	2
Total	295	183	164	161	151	98	76

**Table 5:** Time series analysis (vascular to respiratory)

Filing year	Blood vessel	Artificial intelligence	Cardiac	Remote	Diabetes	Application	Respiratory organs
2004	14	14	14	0	0	14	0
2013	0	0	0	1	0	3	0
2014	3	0	4	0	1	4	0
2015	7	1	4	1	3	4	2
2016	10	3	4	2	3	1	0
2017	11	10	6	4	7	3	7
2018	6	14	5	8	8	7	3
2019	19	24	13	14	3	3	13
2020	7	11	4	4	6	3	2
2021	2	0	3	1	1	0	1
Total	66	64	44	35	32	29	28

**Table 6:** Time series analysis (ophthalmology to dosing).

Filing year	Ophthalmology	Reproductive organ	Big data	Addictive behavior	Dentistry	VRAR	Cancer/tumors	Breast
2009	0	0	0	0	0	0	0	0
2014	0	1	2	1	0	0	0	0
2015	3	2	1	4	1	0	0	1
2016	0	0	0	0	0	0	0	1
2017	3	3	4	3	0	1	3	1
2018	5	13	1	5	4	5	1	2
2019	12	2	7	2	3	3	3	1
2020	5	2	5	0	3	2	3	1
Total	28	23	20	15	11	11	10	7

**Table 7:** Time series analysis (robot to pain).

Filing year	Robot	Gaming	Genetic	Digestive Organs	Obesity	Diet	Allergy	Infection	Pain
2016	1	0	0	2	1	1	0	0	0
2017	0	1	0	0	1	1	1	0	0
2018	3	0	2	0	0	0	1	1	1
2019	2	4	1	1	1	0	1	2	0
2020	0	0	1	1	1	1	0	0	0
2021	0	0	1	0	0	0	0	0	0
Total	6	5	5	4	4	3	3	3	1

#### 3.2.3 Citation analysis of Japanese patent applications

Tables 8, 9, 10 are tables showing the citation analysis in chronological order, based on the number of citations for each major category keyword.

**Table 8:** Citation analysis (medical images to apps).

Filing year	Medical imaging	Central nervous system and psychiatry	Prediction	Biodata	Blood vessel	Cardiac	Sensor	Artificial intelligence	Apps
2004	0	0	0	0	14	14	0	14	14
2006	1	0	0	0	0	0	0	0	0
2007	0	0	0	0	0	0	4	0	0
2009	1	0	0	0	0	0	0	0	0
2011	2	0	2	0	0	0	0	0	0
2012	3	1	0	0	0	0	5	0	0
2013	14	0	0	3	0	0	4	0	1
2014	22	8	5	4	1	4	9	0	3
2015	23	17	17	20	11	8	1	4	1
2016	6	8	4	2	1	1	0	3	0
2017	5	2	2	1	2	2	3	2	0
2018	1	3	3	1	0	0	0	0	2
2019	1	0	0	0	1	1	0	0	0
2020	1	0	0	0	0	0	0	0	0
Total	80	39	33	31	30	30	26	23	21

**Table 9:** Citation analysis (sleep to addictive behavior).

Filing year	Sleep	Image processing	Reproductive organ	Diabetes	Respiratory organs	Remote	Addictive behavior
2012	1	1	0	0	0	0	0
2013	0	1	0	0	0	2	0
2014	3	3	0	1	0	0	0
2015	9	1	4	2	4	1	1
2016	4	2	0	0	0	0	0
2017	0	2	1	2	1	0	0
2018	1	1	1	0	0	1	2
2019	0	1	0	0	0	0	0
Total	18	12	6	5	5	4	3

**Table 10:** Citation analysis (big data to dosing).

Filing year	Big Data	Breast	Gaming	Allergy	VRAR	Ophthalmology	Dosing
2014	1	0	0	0	0	0	0
2015	0	2	0	0	0	0	0
2017	1	0	1	1	1	1	0
Total	2	2	1	1	1	1	1

The highest number of citations was for medical imaging (80 citations), followed by central nervous system/psychiatry (39 citations), prediction (33 citations), biodata (31 citations), vascular (30 citations), cardiac (30 citations), sensor (26 citations), artificial intelligence (23 citations), apps (21 citations), and sleep (18 citations). Imaging had (12 citations), genitourinary (6 citations), diabetes (5 citations), respiratory (5 citations), remote (4 citations), addiction (3 citations), big data (2 citations), breast (2 citations), gaming (1 citation), allergies (1 citation), AR/VR (1 citation), ophthalmology (1 citation), and medication (1 citation). There were no citations for other categories. In most cases, patents from around 2004 were cited, but patents filed after 2012, when the number of patent applications rapidly increased, were also cited.

#### 3.2.4 Number of citations per patent application

Table 11 shows the number of citations per patent application in Japan, with major term keywords as follows: Application (0.72), cardiac (0.68), blood vessel (0.45), artificial intelligence (0.36), allergy (0.33), breast (0.29), medical image (0.27), reproductive (0.26), central nervous system and psychiatry (0.24), bio‐data (0.21), addictive behavior (0.20), gaming (0.20), sleep (0.18), prediction (0.18), respiratory organs (0.18), dosing (0.17), sensor (0.16), image processing (0.16), diabetes (0.16), remote (0.11), big data (0.10), vr/ar (0.09), and ophthalmology (0.04).

**Table 11:** Number of citations per patent application in Japan.

Main terms	Ratio of citations/application
Application	0.72
Cardiac	0.68
Blood vessel	0.45
Artificial intelligence	0.36
Allergy	0.33
Breast	0.29
Medical image	0.27
Reproductive	0.26
Central nervous system and psychiatry	0.24
Biodata	0.21
Addictive behavior	0.2
Gaming	0.2
Sleep	0.18
Prediction	0.18
Respiratory organs	0.18
Dosing	0.17
Sensor	0.16
Image processing	0.16
Diabetes	0.16
Remote	0.11
Big data	0.1
VRAR	0.09
Ophthalmology	0.04

### 3.3 Patent technology analysis

We conducted a patent technology analysis on the top most cited Japanese patent applications by category (major term keywords). The patent technology analysis is conducted by referring to the applicant, the name of the invention, and the abstract (the source is the gazette number).

#### 3.3.1 Patent technology analysis

(1) Japanese Patent Application No.: 2006‐552 106.

(Number of citations: 14, Keywords: Blood Vessel, Artificial Intelligence, Heart, App.)

[Applicant] Siemens Medical Solutions USA Incorporated, Siemens Corporation.

[Title of invention] System and method for automated diagnosis and decision support for heart‐related diseases and conditions.

[Summary]

…to provide decision support for various aspects of a physician's workflow, including automated diagnosis of conditions such as heart‐related medical conditions……a CAD (computer‐aided diagnosis) system for cardiac imaging and applications are provided.

(2) Japanese patent application No.: 2015‐249 842

(Number of citations: 5, Keyword: prediction)

[Applicant] Panasonic Corporation

[Title of the Invention] Stimulus presentation system, method of stimulus presentation, computer, and control method

[Summary]

[Problem] To provide a stimulus presentation system that leads the user's psychological state to the target psychological state.

[Solution] The stimulus presentation system of the present disclosure is equipped with… a psychological state determination unit that determines whether or not the user has reached the target psychological state… based on the user's biometric information acquired after the presentation of the stimulus presentation contents has started.

(3) Japanese patent application No.: 2014‐263 875

(Number of citations: 4, Keyword: sensor)

[Applicant] Unicharm Corporation

[Title of the Invention] Programs used for childcare support, childcare support methods, childcare support systems, and infant sensor devices

[Summary]

…at least two of the three types of processing to evaluate the infant's temperature, heart rate, and how often the infant's diaper is changed, and…to display a main icon with a main indicator of the degree of good health of the infant…The program causes the infant to perform the following processes….

(4) Japanese Patent Application No.: 2016‐548612

(Number of citations: 4, Keywords: central nervous system, mental)

[Applicant] Sony Corporation

[Title of the Invention] State Control Equipment, State Control Method, and State Control System

[Summary]

The biometric information includes information indicating a detection result of an enzyme. The biometric information includes information indicating a detection result of an enzyme.

(5) Japanese patent application No.: 2015‐100095

(Number of citations: 3, Keywords: sleep, central nervous system/psychiatric)

[Applicant] Econavista Corporation

[Title of the Invention] Information process equipment, programs, information processing methods, and information processing systems

[Summary]

Provide information processing equipment that easily evaluates the degree of recovery from fatigue before and after sleep….

(6) Japanese Patent Application No.: 2016‐556 748

(Number of citations: 3, Keywords: respiratory, vascular, heart)

[Applicant] Koninklijk Reckers Philips N.V.

[Title of invention] Patient monitoring and therapeutic intervention/event timeline

[Summary]

…Vital signs include blood pressure (BP), blood oxygen (SpO2), heart rate (HR), and respiratory rate (RR). Receive monitored vital signs and determine a triage score based on the vital signs received, the subject's gender, the subject's age, and the subject's symptoms.

(7) Japanese Patent Application No.: 2015‐14406

(Number of citations: 4, Keywords: reproductive organs, biological data)

[Applicant] Nomura Research Institute, Ltd.

[Title of the Invention] Health Care System

[Summary]

The health care system registers and manages health information including test result data, body temperature, and menstrual data for each user, determines the health status of each user, and outputs information including a graph of test result data, a graph of body temperature and menstrual data, and a message corresponding to the health status of each user to the user's terminal 2. Output information including a graph of test result data, a graph of temperature and menstrual data, and a message according to the health status of each user to the user's terminal 2.

(8) Japan Patent Application No. 2017‐83342

(Number of citations: 2, Keywords: diabetes, blood vessel, heart, sensor)

[Applicant] VIAVI Solutions, Inc.

[Title of the Invention] Health Tracking Device

[Summary]

…The device has multiple types of sensors, including a spectrometer and one or more of the following: accelerometer, heart rate sensor, blood pressure sensor, blood glucose sensor, sweat sensor, skin conduction sensor, or image sensor. The device processes… sensor data to determine the health status of the user.

(9) Japanese Patent Application No.: 2015‐82925

(Number of citations: 2, Keyword: breast)

[Applicant] Canon Medical Systems Inc.

[Title of the Invention] Medical Information Processing System

[Summary]

…A medical information processing system that manages mammography examinations and…calculates a mammary gland density index, which represents the degree of mammary gland density, based on mammography images.

(10) Japanese patent application No.: 2009‐148070

(Number of citations: 1, Keyword: medication)

[Applicant] Toshiro Majima

[Title of the Invention] Method of Health Care Differentiation and Indication of the Evaluation thereof

[Summary]

…It integrates nutritional status, disease status, and psychosomatic functional status, and provides a method for differentiating health status and health status orientation. The method divides the situation into (1) the state of the individual's physical and mental customs and (2) the pathological situation, assigns a positive grade if the situation is in accordance with the health orientation and a negative grade if it is contrary to it, and differentiates… by the sum of the calculated grades.

### 3.4 Summary of result

The results of the analysis of the number of patent applications, the number of citations, the number of citations per patent application, and the patented technologies are shown in Table 12. According to Table 12, the key drivers Medical Image, Prediction, Sensors, Central Nervous System and Psychiatry, and Bio‐data are identified based on the number of patent applications. The key drivers Medical Image, Central Nervous System and Psychiatry, Prediction, Bio‐data, Vascular, and Cardiac are identified based on the number of citations. The key drivers Application, Cardiac, and Vascular are identified based on the number of citations per patent application. Technologies, applications, and user interfaces that analyze medical images and patient data, leading to diagnostic evaluations and target conditions, are identified as the key drivers based on the patented technologies.

**Table 12:** Key drivers based on the patent analysis.

Method of analysis	Key drivers
Number of patent applications	Medical image/prediction/sensors/central nervous system and mental/biodata
CI	Medical imaging/central nervous system and mental/prediction/bio data/vascular/cardiac
Number of citations per patent application	Application/cardiac/vascular
Patented technology	Technologies, applications, and user interfaces that analyze medical images and patient data and lead to diagnostic evaluations and target conditions

A previous study analyzed patents filed with the United States Patent and Trademark Office and calculated the score of potential technologies based on their thematic characteristics with respect to their digital capabilities and similarity to DTx technologies. The study suggested that technologies using sensors to monitor patients' sleep and breathing patterns or specifically symptoms related to environmental factors are potential targets for digital therapeutics.^3^

Another previous report researched the potential to pursue digital use‐cases and analyzed the DTx ecosystem in terms of therapeutic areas (endocrine, central nervous system (cns), cardiovascular, respiratory, oncology, immunology) and digital capabilities (data tracking, predictive analytics and algorithms, communications and social media, sensor and location services, gaming and virtual reality, artificial intelligence and machine learning) as highly potential markets for digital therapeutics.^4^

## 4 DISCUSSION

IPC “G16H” and “A61B” are assigned in the medical information field. Patent applications have been increasing since 2015 worldwide and since around 2012 in Japan. In the field of medical information, Japan has been one of the first countries in the world to file patent applications. Additionally, Japan is considered to have been actively engaged in R&D in the medical information field. However, in terms of recent numbers of patent applications, many applications are filed in the United Staes, China, and Korea, indicating that R&D is being conducted in these regions or that these regions are considered valuable as markets. Major electronics manufacturers were the top applicants/right holders in the medical information field.

From the details of the IPC, the keywords “computing,” “patient data,” and “diagnostic radiology equipment” were found as applicable technologies, and “diagnosis” and “evaluation” as objectives.

Furthermore, in the medical information field, narrowing down the keywords in terms of applicable technologies and indications, in Japan, there were many patent applications corresponding to applicable technologies and indications related to “medical images,” “prediction,” “sensors,” “central nervous system/mental,” and “biological data.” It can be assumed that research and development in the medical information field are actively conducted in these applicable technologies and indications.

In terms of the number of citations in the medical information field, it is considered that in Japan, a large number of highly valuable patent applications are filed in the fields of “medical imaging,” “central nervous system/mental,” “prediction,” “biological data,” “vascular,” and “cardiac.”

Based on the number of citations per patent application in the medical information field, it can be assumed that in Japan, “apps,” “cardiac,” and “vascular” are most common, and the value per patent application is high in these applicable technologies and indications, and competition is intense.

As a result of the analysis of patent technologies with a large number of citations in the medical information field, in Japan, “technologies and applications related to technologies and user interfaces that analyze medical images and patient data and lead to diagnostic evaluation and target conditions” have a large number of citations and are considered to have high value. The technologies developed in Japan focus on data and user interfaces.

However, in the medical information field, there have been few patent applications for digital therapeutics for the direct purpose of disease prevention, diagnosis, and treatment, and they have been buried in many patent applications, making it difficult to distinguish between digital therapeutics and other medical information technologies.

## 5 CONCLUSION

Key drivers of digital therapy were examined by analyzing patents in the fields of healthcare informatics and diagnosis (medical information field), using the applicable technologies and indications as key drivers.

In terms of the number of patent applications and citations in Japan, there were many patents related to “applications,” “sensors,” “medical imaging,” “central nervous system/psychiatry,” and “cardiac,” indicating a common need in Japan. Therefore, Japanese companies are expected to conduct R&D with an eye to overseas expansion.

We suggest that Japanese companies have significant advantages and should utilize these technologies for DTx of the target disorders and pursue digital use‐cases in the area as highly potential markets of DTx.

The data that support the findings of this study are available from the corresponding author upon reasonable request.

## AUTHOR CONTRIBUTIONS

**Tetsuaki Oda**: Conceptualization; funding acquisition; methodology; project administration; writing—original draft. **Chikako Oda**: Conceptualization; data curation; formal analysis.

## CONFLICTS OF INTEREST STATEMENT

The authors, whose names are listed immediately below, certify that they have no affiliations with, or involvement in, any organization or entity with any financial interest (such as honoraria, educational grants, participation in speakers' bureaus, membership, employment, consultancies, stock ownership, or other equity interest, and expert testimony or patent‐licensing arrangements) or nonfinancial interest (such as personal or professional relationships, affiliations, knowledge, or beliefs) in the subject matter or materials discussed in this manuscript.

## ETHICS STATEMENT

This manuscript has not been previously published and is not under consideration for publication elsewhere. We have adhered to ethical principles throughout the research process, including the appropriate use of data and citation of sources.

## TRANSPARENCY STATEMENT

The lead author Tetsuaki Oda affirms that this manuscript is an honest, accurate, and transparent account of the study being reported; that no important aspects of the study have been omitted; and that any discrepancies from the study as planned (and, if relevant, registered) have been explained.

## Data Availability

The data that support the findings of this study are available from the corresponding author upon reasonable request.

## References

[1] PatentSQUARE . https://www.panasonic.com/jp/business/its/patentsquare.html

[2] Oda T , Gemba K , Matsushima K . Enhanced co‐citation analysis using frameworks. Technol Anal Strat Manage. 2008;20(2):217‐229.

[3] Jeon E , Yoon N , Sohn SY . Exploring new digital therapeutics technologies for psychiatric disorders using BERTopic and PatentSBERTa. Technol Forecase Soc. 2023;186:122130.

[4] *Deloitte, Digital therapeutics Improving Patient Outcomes Through Convergence*; 2019. https://www2.deloitte.com/us/en/pages/life-sciences-and-health-care/articles/digital-therapeutics.html

